# Temporal patterns in diabetes and sepsis mortality in older Americans: a population-based analysis

**DOI:** 10.1186/s12877-026-06989-8

**Published:** 2026-01-26

**Authors:** Talha Ali, Laksh Kumar, Faiqa Iqbal, Syed Hussain Ahmed Rizvi, Muhammad Abdullah Naveed Uz Zafar, Muhammad Ahmed, Bazil Azeem, Muhammad Abdullah Naveed, Himaja Dutt Chigurupati, Binish Qureshi, Harigopal Sandhyavenu, Sivaram Neppala

**Affiliations:** 1 Department of Medicine, Shaheed Mohtarma Benazir Bhutto Medical College Lyari, Karachi, Pakistan; 2 Department of Medicine, Dow Medical College, Dow University of Health Sciences, Karachi, Pakistan; 3 Division of Cardiology, East Carolina University, Greenville, NC USA; 4 Division of Cardiology, The University of Texas Health Sciences Center, San Antonio, TX USA

**Keywords:** Diabetes, Sepsis, Mortality rate, Epidemiology, Demographics, Healthcare disparities

## Abstract

**Background:**

Diabetes mellitus significantly elevates the risk of sepsis and exacerbates its clinical outcomes, leading to increased mortality rates. Despite the importance of this association, the demographic and geographic patterns influencing it remain inadequately understood.

**Methods:**

Utilizing data from CDC WONDER (1999–2020), we conducted an analysis of mortality trends among U.S. adults aged 65 and older, stratified by gender, race/ethnicity, and geographic region. Age-adjusted mortality rates (AAMRs) and annual percentage changes (APCs) were evaluated using the Joinpoint Regression methodology.

**Results:**

Between 1999 and 2020, there were a total of 287,738 deaths attributed to diabetes and sepsis. The AAMR decreased from 33.6 per 100,000 population in 1999 to 27.7 in 2014; however, it subsequently surged to 38.4 by 2020 (APC: 13.65). The male AAMRs reached a peak of 39.1 in 2005, declined to 31.2 in 2012, and then increased to 46.9 in 2020 (APC 12.29). Female AAMRs decreased from 32.6 in 1999 to 24.8 in 2018, before rising again to 31.9 in 2020 (APC 13.66). Among African Americans, mortality declined to 46.3 in 2019 but experienced a sharp increase to 65.7 in 2020 (APC 20.52). The Hispanic AAMR also increased during this period (APC 25.05). The southern region consistently exhibited the highest mortality rates, rising from 31.7 in 2018 to 42.5 in 2020 (APC 13.69), with notable rises observed in the Midwest (APC 14.59). All these trends were statistically significant (*p* < 0.05). Furthermore, non-metropolitan areas consistently demonstrated higher mortality rates, with significant increases observed between 2018 and 2020.

**Conclusion:**

The mortality rates associated with diabetes-related sepsis have recently risen, particularly between 2018 and 2020, thereby highlighting significant health disparities. It is essential to implement targeted healthcare interventions.

**Supplementary Information:**

The online version contains supplementary material available at 10.1186/s12877-026-06989-8.

## Introduction

Diabetes mellitus is a chronic medical condition characterized by hyperglycemia resulting from impaired insulin function. Its global prevalence is increasing, with projections indicating a rise from 415 million cases in 2015 to 642 million by 2040, predominantly in low- and middle-income countries. In 2013, diabetes was responsible for 5.1 million deaths, accounting for approximately 8.4% of all-cause mortality among adults aged 20 to 79 years [1, 2]. Type 1 diabetes results from autoimmune destruction of pancreatic beta cells, whereas type 2 diabetes is caused by insulin resistance and beta cell dysfunction [3]. Sepsis, a life-threatening condition caused by an uncontrolled immune response to infection, is a significant contributor to worldwide mortality. It affects an estimated 47–49 million individuals annually, leading to roughly 11 million deaths, which constitutes approximately 20% of global mortality [4, 5]. Additionally, diabetes increases the risk of developing sepsis and aggravates its complications, with both conditions frequently co-occurring, thereby complicating their individual and combined impacts on health outcomes [6].

This study aims to examine and describe trends in the combined effects of diabetes and sepsis on mortality across diverse demographic and regional populations within the United States. Utilizing data from the Centers for Disease Control and Prevention’s Wide-ranging ONline Data for Epidemiologic Research (CDC-WONDER) database, the research endeavors to elucidate the interactions and influences of these diseases on mortality patterns. Specifically, the investigation will analyze regional disparities and demographic variations in mortality rates, thereby addressing existing gaps in the literature and offering valuable insights for targeted public health strategies.

## Methodology

### Data source

To investigate mortality trends among older adults with diabetes and sepsis, we employed data from the CDC WONDER database [7]. The analysis concentrated on records classified according to the International Classification of Diseases, Tenth Revision (ICD-10), with codes A40-A41 for sepsis and E10-E14 for diabetes. Death certificates were scrutinized to determine whether sepsis and diabetes were identified as either contributing or underlying causes of death. Institutional review board approval was deemed unnecessary, as the analysis utilized publicly accessible data.

### Data extraction

Data were extracted from the CDC WONDER database for all fifty U.S. states spanning from 1999 to 2020. The dataset encompassed demographic information, population size, year of death, and geographical location. Demographic variables included age (≥ 65 years), gender (male and female), race, and ethnicity, with the following categories specified: Non-Hispanic White, Non-Hispanic Black or African American, Hispanic or Latino, Non-Hispanic American Indian or Alaska Native, and Non-Hispanic Asian or Pacific Islander. Geographical classifications followed the Urban-Rural Classification Scheme for Counties by the National Center for Health Statistics, categorizing areas into large metropolitan (with ≥ 1 million inhabitants), medium/small metropolitan (with 50,000–999,999 inhabitants), and rural (with < 50,000 inhabitants) categories [8]. Furthermore, regions were grouped into Northeast, Midwest, South, and West to facilitate a comprehensive analysis of mortality trends across diverse population groups and geographic locations.

### Data analysis

We analyzed mortality patterns for sepsis and diabetes from 1999 to 2020, examining trends based on race, ethnicity, place of death, urbanization, and census regions. Both crude mortality rates, calculated by dividing the total number of deaths due to sepsis and diabetes by the corresponding U.S. population, and age-adjusted mortality rates (AAMR) per 100,000 individuals were computed. The AAMR was standardized using the 2000 U.S. standard population [9].

In the sensitivity analysis, we restricted the dataset to deaths where sepsis was explicitly listed as the primary cause. This enabled us to isolate mortality directly attributable to sepsis among individuals with diabetes and compare it with overall diabetes-sepsis mortality patterns. The summary results of this analysis are presented in Supplementary Table 8. Temporal trends were assessed utilizing the Joinpoint Regression Program (Version 5.0.2, National Cancer Institute), which employs log-linear regression models to fit a series of joined straight lines on a logarithmic scale [10]. This approach yielded the annual percentage change (APC) in the average annual mortality rate (AAMR), along with its 95% confidence interval (CI). Mortality trends were categorized as increasing or decreasing based on whether the slope of the trend was significantly different from zero. Furthermore, a two-tailed t-test was employed to evaluate whether the slope of the annual percent change demonstrated a significant deviation from zero, with a *P*-value of < 0.05 regarded as indicative of statistical significance.

## Results

### Overall mortality trends

**Fig. 1:**
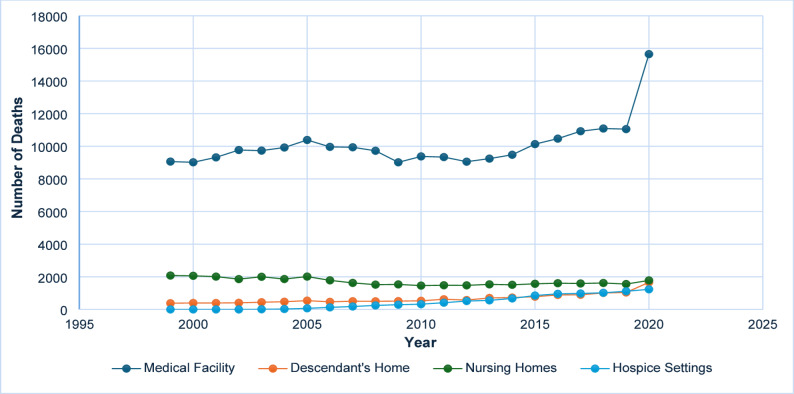
Number of deaths due to diabetes and sepsis by place of death, 1999–2020

Between 1999 and 2020, a total of 287,738 deaths associated with diabetes and sepsis were recorded among U.S. adults aged 65 years and older. Of these, 134,110 were males and 153,628 were females. Data regarding the place of death were available for 283,280 individuals, with 22,168 succumbing in medical facilities, 14,463 in the homes of descendants, 9,584 in hospice settings, and 3,754 in nursing homes. These data were included to illustrate primary settings in which diabetes-related sepsis deaths occurred, thereby providing contextual insight into the acute nature of these events and the healthcare environments most affected **(**Fig. 1 and Supplementary Table 1).

### General mortality trends

**Fig. 2:**
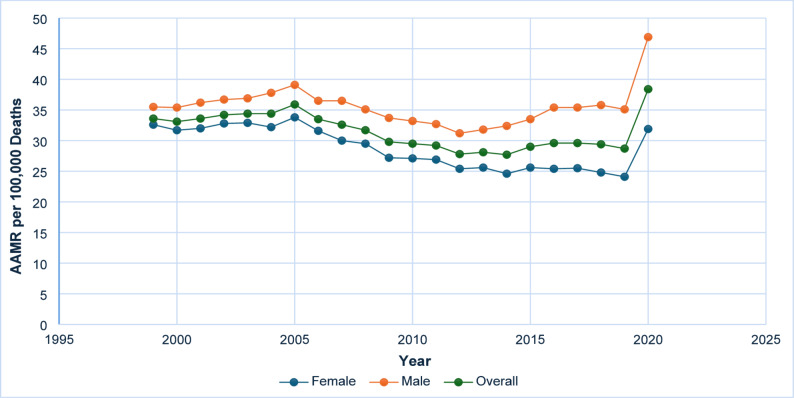
AAMR-related to diabetes and sepsis stratified by sex/gender, 1999–2020

From 1999 to 2020, the mortality rate attributable to diabetes and sepsis among United States adults demonstrated a dynamic trend. The AAMR was 33.6 per 100,000 population (95% CI: 33.0–34.2) in 1999, followed by a period of stability, after which it gradually declined to 27.7 (95% CI: 27.2–28.2) by 2014. Between 1999 and 2018, the AAMR exhibited a statistically significant annual percent change (APC) of − 1.19* (95% CI: −2.18 to − 0.67; *p* < 0.05), indicating a consistent downward trend. However, between 2018 and 2020, a notable increase was observed, with the AAMR rising from 29.4 (95% CI: 29.0–29.9) in 2018 to 38.4 (95% CI: 37.9–38.9) in 2020. This transition corresponded to a significant APC of 13.65* (95% CI: 1.35 to 19.77; *p* = 0.05), potentially reflecting the influence of emerging challenges within the healthcare landscape during this period **(**Fig. 2 and Supplementary Table 2, Supplementary Table 7).

### Gender differences

Between the years 1999 and 2020, mortality rates associated with diabetes and sepsis exhibited notable differences between males and females. Among males, the AAMR increased from 35.5 per 100,000 population (95% Confidence Interval [CI]: 34.5–36.6) in 1999 to a peak of 39.1 (95% CI: 38.1–40.1) in 2005, subsequently declining gradually to 31.2 (95% CI: 30.4–32.1) by 2012. During the period from 1999 to 2005, the APC was 1.42 (95% CI: −4.46 to 11.52), followed by a non-significant decrease from 2005 to 2012 with an APC of − 2.75 (95% CI: −7.96 to 5.82). After a modest increase from 2012 to 2018, indicated by an APC of 1.91 (95% CI: −3.71 to 5.27), mortality rates rose sharply from 35.8 (95% CI: 35.0–36.6) in 2018 to 46.9 (95% CI: 46.1–47.8) in 2020, reflecting a significant APC of 12.29 (95% CI: 3.49 to 17.69; *p* < 0.05). Conversely, females experienced a consistent decline in mortality from 32.6 (95% CI: 31.8–33.4) in 1999 to 24.8 (95% CI: 24.2–25.4) in 2018, with a significant APC of − 1.88 (95% CI: −2.47 to − 1.41; *p* < 0.05). Nonetheless, from 2018 to 2020, the Age-Adjusted Mortality Rate increased markedly to 31.9 (95% CI: 31.3–32.6), corresponding to a significant APC of 13.66 (95% CI: 3.42 to 18.71; *p* < 0.05). These trends indicate a recent reversal of long-term declines in mortality, notably rapid among both sexes during the period from 2018 to 2020 **(**Fig. 2 and Supplementary Table 2, Supplementary Table 7).

### Racial and ethnic differences

**Fig. 3:**
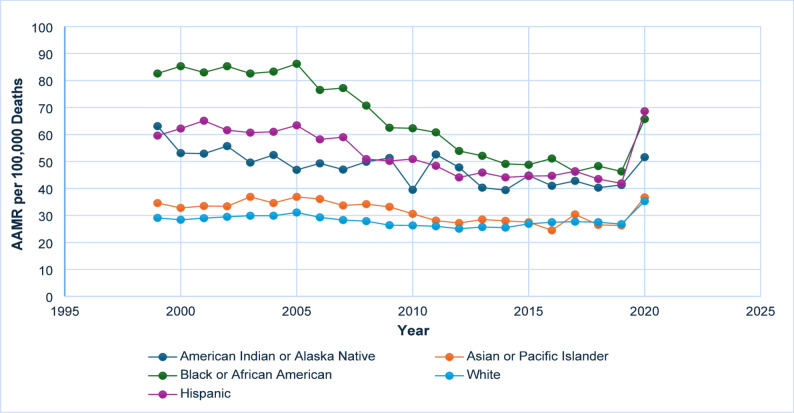
AAMR-related to diabetes and sepsis stratified by race/ethnicity, 1999–2020

Black or African American individuals consistently experienced the highest AAMRs throughout the study period. The AAMR began at 82.6 (95% CI: 79.2–86.0) in 1999, showing a steady and significant decline to 46.3 (95% CI: 44.4–48.3) by 2019, followed by a sharp increase to 65.7 (95% CI: 63.4–67.9) in 2020. A significant decline was observed between 2004 and 2018 (APC: −4.66*; 95% CI: −6.37 to − 4.06; *p* < 0.05), followed by a notable increase from 2018 to 2020 (APC: 20.52; 95% CI: 9.56 to 27.79; *p* < 0.05). The line graph for racial and ethnic differences is provided in Fig. 3. Data is tabulated in Supplementary Tables 3 and Supplementary Table 7.

#### White (non-hispanic)

White individuals exhibited the lowest overall AAMRs among non-Hispanic groups, demonstrating a consistent decline from 29.1 (95% CI: 28.5–29.7) in 1999 to 26.8 (95% CI: 26.3–27.3) in 2019, followed by an increase to 35.3 (95% CI: 34.8–35.9) in 2020. The long-term trend from 1999 to 2018 indicated a statistically significant decrease (APC: −0.80*; 95% CI: −1.99 to − 0.26), whereas the increase observed during 2018–2020 was also statistically significant (APC: 13.84; 95% CI: 1.41 to 20.24).

#### Hispanic or Latino

Individuals of Hispanic or Latino descent initially demonstrated elevated mortality rates, with an AAMR of 59.6 (95% CI: 55.6–63.5) in 1999. Following a persistent and notable decline through 2018 (APC: −2.47*; 95% CI: −3.38 to − 1.68), the rates subsequently increased to 68.6 (95% CI: 66.2–71.1) by 2020. This represented a significant APC of 25.05 (95% CI: 10.46 to 32.98).

#### American Indian or Alaska native

Mortality rates fluctuated but generally declined from 63.1 (95% CI: 50.2–78.3) in 1999 to 41.3 (95% CI: 34.9–47.7) in 2019, before increasing to 51.6 (95% CI: 44.8–58.5) in 2020. A significant decline was observed from 1999 to 2018 (APC: −1.81*; 95% CI: −4.24 to − 0.87), followed by a non-significant rise from 2018 to 2020 (APC: 11.73; 95% CI: −0.94 to 18.60).

#### Asian or Pacific Islander

Individuals of Asian or Pacific Islander descent experienced relatively low mortality rates throughout the period, beginning at 34.6 (95% CI: 30.1–39.0) in 1999 and increasing to 36.7 (95% CI: 34.4–39.1) in 2020. A statistically significant decline was observed from 1999 to 2018 (APC: −1.90*; 95% CI: −3.69 to − 0.96), with a notable reversal during 2018–2020 (APC: 15.38*; 95% CI: 0.47 to 23.10).

### Regional differences

**Fig. 4:**
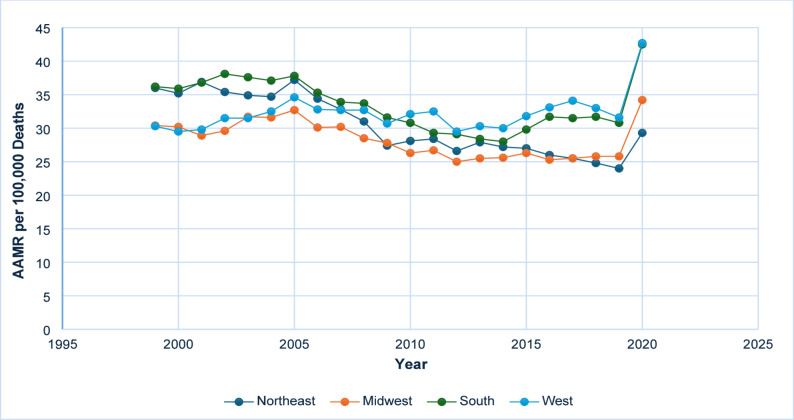
AAMR-related to diabetes and sepsis stratified by census/region, 1999–2020

From 1999 to 2020, the Southern region consistently exhibited the highest average AAMR for diabetes and sepsis-related mortalities, followed by the Northeast, West, and Midwest regions. The results of the line graph for regional differences are provided in Fig. 4 and Supplementary Table 4.

#### Southern Region

The AAMR remained relatively high and stable from 1999 (36.2) to 2005 (37.8), with a non-significant APC of 0.50 (95% CI: -4.67 to 9.49). A statistically insignificant decline was observed between 2005 and 2012, with an APC of − 3.88 (95% CI: −9.39 to 4.40). From 2012 to 2018, mortality rates exhibited a modest, non-significant increase, with an APC of 1.52 (95% CI: −4.31 to 5.21). However, between 2018 and 2020, the region experienced a noteworthy and statistically significant increase in mortality rates, rising from 31.7 to 42.5, with an APC of 13.69 (95% CI: 3.87 to 19.64; *p* < 0.05).

#### Northeast Region

An initial modest increase from 1999 (36.0) to 2005 (37.2), characterized by an APC of 0.25 (95% CI: −2.86 to 3.70), was subsequently followed by a decline between 2005 and 2009, with an APC of − 5.47 (95% CI: −8.16 to 2.10). From 2009 to 2018, mortality continued its downward trend, albeit at a more gradual pace, with an APC of − 1.62 (95% CI: −5.09 to 0.05). In the final period from 2018 to 2020, mortality increased from 24.8 to 29.3, with an APC of 5.98 (95% CI: −0.79 to 9.57); however, this change was not statistically significant.

#### Midwest Region

The AAMR experienced a slight increase from 1999 (30.4) to 2005 (32.7), with an APC of 1.41 (95% CI: −0.33 to 5.06), followed by a decline through 2010 (APC: −3.80; 95% CI: −7.13 to 0.21). From 2010 to 2018, the trend remained stable, with a non-significant APC of − 0.69 (95% CI: −2.49 to 1.86). A pronounced and statistically significant rise was observed between 2018 and 2020, with the AAMR increasing from 25.8 to 34.2, corresponding to an APC of 14.59* (95% CI: 7.12 to 19.01; *p* < 0.05).

#### Western Region

The Western region exhibited a relatively stable AAMR from 1999 (30.3) to 2018 (33.0), with an almost negligible Annual Percent Change (APC) of 0.12 (95% Confidence Interval: -0.76 to 0.67). A noteworthy increase was observed between 2018 and 2020, during which the AAMR increased from 33.0 to 42.7, corresponding to an APC of 12.46* (95% Confidence Interval: 2.50 to 17.61; *p* < 0.05).

### Urbanization

**Fig. 5:**
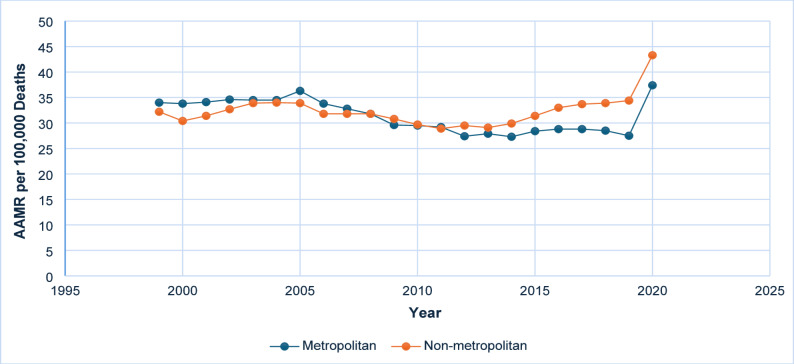
AAMR-related to diabetes and sepsis stratified by urbanization, 1999–2020

Between 1999 and 2020, non-metropolitan (rural) regions consistently demonstrated higher average AAMRs for deaths related to diabetes and sepsis in comparison to metropolitan (urban) areas. A noteworthy rise was recorded in both rural and urban regions during the period from 2018 to 2020 **(**Fig. 5 and Supplementary Table 5, Supplementary Table 7).

### States

**Fig. 6:**
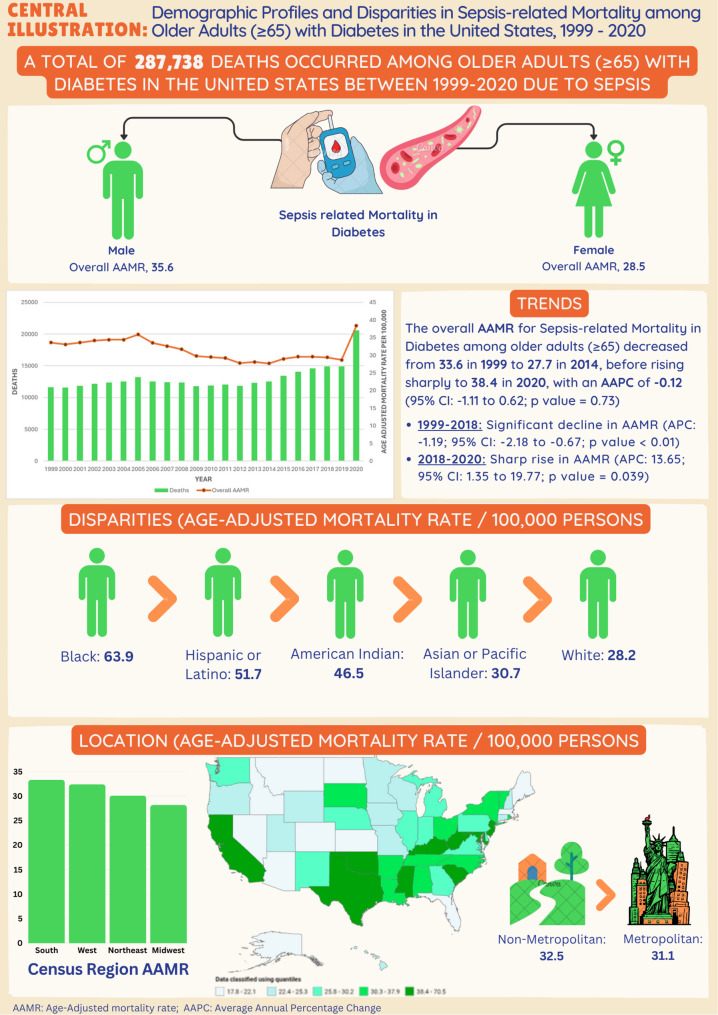
The central illustration depicts trends in demographics and disparities in mortality related to diabetes mellitus among sepsis patients in the United States from 1999 to 2020

From 1999 to 2020, states in the top 90th percentile for age-adjusted mortality included the District of Columbia with an AAMR of 70.5 (66.3–74.6), Oklahoma at 49.7 (48.4–51.0), Texas at 45.9 (45.3–46.4), Kentucky at 45.2 (44.0–46.4), Maryland at 42.6 (41.6–43.7), and California at 42.0 (41.6–42.5). These states exhibited consistently elevated mortality rates over the study period. In contrast, states in the bottom 10th percentile included Arizona with an AAMR of 17.8 (17.3–18.4), Florida at 17.8 (17.5–18.1), Alaska at 19.5 (16.8–22.1), Maine at 19.6 (18.3–20.8), Colorado at 21.6 (20.8–22.4), and Idaho at 20.2 (18.9–21.5), reflecting substantially lower mortality burdens (Fig. 6 and Supplementary Table 9).

## Discussion

This retrospective study examines the trends in mortality associated with diabetes and sepsis in the United States from 1999 to 2020, highlighting the impact of diverse demographic and regional factors. The data demonstrate an overall decline in mortality rates from 1999 to 2018, followed by a significant increase from 2018 to 2020. Gender-specific analysis disclosed distinct mortality patterns for men and women, with a notable elevation in male mortality during the final period. Regional disparities consistently indicated elevated mortality rates in the Southern region, coupled with evident racial and ethnic disparities, particularly among African Americans and Hispanics. Furthermore, non-metropolitan areas exhibited higher mortality rates compared to urban regions, with a sharp rise in both settings between 2018 and 2020.

For men, mortality rates peaked in 2005 and gradually declined until 2012, followed by a significant rise from 2018 to 2020. This fluctuation may be attributed to gender-specific health factors, such as a higher prevalence of comorbidities and lifestyle-related conditions in men. Furthermore, biological differences, including hormonal variations, may play a role, as men typically experience higher rates of chronic conditions like cardiovascular diseases [11, 12]. Additionally, men generally have lower healthcare utilization, which can lead to delayed diagnoses and the worsening of health conditions, thereby exacerbating these disparities [11, 13]. Conversely, for women, mortality rates consistently declined until 2018, followed by a resurgence in subsequent years, mirroring the trends observed in men. This pattern indicates that societal or healthcare disruptions, including diminished access to medical services or the exacerbating effects of the COVID-19 pandemic, likely affected both genders similarly [11, 14].

The significant rise in mortality rates among African Americans from 2018 to 2020, following years of decline, underscores enduring inequities in healthcare access, chronic disease management, and socioeconomic conditions. African Americans historically experience a higher prevalence of diabetes and infection-related complications, which may be compounded by systemic barriers to timely care and disparities in healthcare resource allocation. These challenges were further magnified during the COVID-19 pandemic, which disproportionately affected communities of color and exacerbated existing inequities in chronic disease management, access to testing, vaccination, and critical care [1, 15, 16]. Similarly, the reversal of declining trends among Hispanic and Latino populations highlights vulnerabilities linked to occupational exposures, health insurance coverage, and access to preventive services [17]. These patterns collectively reflect the intersection of biological susceptibility and social determinants of health, warranting targeted public health interventions.

The Southern region consistently demonstrated the highest average mortality rates throughout the study period. This trend is attributable to the region’s healthcare infrastructure, socioeconomic conditions, and a higher prevalence of obesity and chronic diseases [18, 19]. Although the Northeastern, Western, and Midwestern regions also experienced increases in mortality rates, the Southern region persistently maintained elevated figures, indicating the existence of systemic challenges that require targeted interventions. Also, this research delineated a concerning trend whereby rural (non-metropolitan) regions persistently exhibited higher mortality rates compared to urban (metropolitan) areas. This disparity was particularly pronounced between 2018 and 2020, potentially indicating delays in healthcare access, a reduced number of healthcare facilities, and lower levels of health literacy in rural areas [18, 20]. Although urban regions initially demonstrated higher mortality rates in 1999, they maintained a relatively stable trend until a recent increase, which may be attributed to factors such as overcrowding, heightened exposure to strain on the healthcare system, or demographic shifts [21, 22]. Addressing these issues requires the implementation of targeted interventions that focus on enhancing healthcare infrastructure and optimizing resource allocation in urban areas.

The pathophysiological factors contributing to the rising mortality rates associated with diabetes and sepsis are multifactorial. Diabetes, a chronic condition that impairs immune function, places individuals at an elevated risk for infections such as sepsis. Sepsis, a life-threatening reaction to infection, is more common in older adults and those with underlying conditions like diabetes [6, 23, 24]. These infections can overwhelm the immune system, particularly in individuals with poorly controlled blood glucose levels. The recent surge in mortality rates, particularly between 2018 and 2020, coincided temporally with the onset of the COVID-19 pandemic. While our ecological design precludes direct attribution, this period overlap suggests a plausible influence of pandemic-related healthcare disruptions, as reported in prior literature [14, 15]. Comorbidities, including cardiovascular diseases and hypertension, are common among diabetic patients, further exacerbating the risks associated with sepsis [25, 26]. The interplay of these conditions creates a vicious cycle, where poor management of one health aspect exacerbates others, resulting in poorer overall outcomes.

To enhance patient outcomes, it is essential to adopt a dual approach that focuses on the prevention and effective management of both diabetes and sepsis. Early detection and intervention for diabetes, achieved through lifestyle modifications, medication, and patient education, are essential. Similarly, optimizing sepsis management requires early identification and swift intervention, including the administration of antibiotics, fluid resuscitation, and supportive care [27–29]. Implementing targeted interventions for high-risk populations such as African Americans, Hispanics, and rural residents, is of vital importance. Expanding healthcare access, particularly in underserved areas, and addressing health disparities through culturally competent care are crucial steps toward reducing mortality rates [20, 30]. Moreover, integrating preventive care strategies, such as routine diabetes screening and infection vaccination, can contribute to lowering the incidence of sepsis-related fatalities.

### Limitations

Despite its valuable contributions, this study is subject to several limitations. The reliance on death certificate data introduces potential inaccuracies due to misclassification or underreporting of underlying causes. In addition, the analysis does not account for individual-level confounders such as comorbidities, disease severity, genetic predisposition, socioeconomic status, and behavioral health factors limits the study’s ability to infer casual pathways or adjust for confounding factors. Therefore, the results should be interpreted as descriptive indicators of temporal and demographic disparities, rather than as evidence of mechanistic relationships. While demographic variables like age, gender, and race were included, the lack of finer stratification limits the capability to discern nuanced trends. Future research should prioritize the collection of detailed, individual-level data to better elucidate the specific causes and risk factors associated with mortality from diabetes and sepsis. Cohort studies incorporating broader variables such as lifestyle factors, healthcare access, and health behaviors, could offer deeper insights into the mechanisms driving these outcomes. Moreover, longitudinal designs are essential to assess the sustained impact of interventions, including diabetes management programs and sepsis treatment protocols, on mortality trajectories. Finally, the decision to aggregate Asian and Pacific Islander populations may obscure important heterogeneity within these groups. A more granular approach that disaggregates these populations is warranted to yield more accurate and culturally sensitive findings.

## Conclusion

From 1999 to 2018, mortality rates due to diabetes and sepsis exhibited a consistent decline within the United States; however, there was a resurgence from 2018 to 2020, which illuminated increasing health disparities. The most significant rise was observed among men, minority populations, rural communities, and residents of the Southern regions. This trend is primarily attributable to unequal access to healthcare resources, the prevalence of chronic conditions, and challenges associated with the pandemic. Addressing this issue necessitates early diagnostic interventions, the provision of equitable healthcare services, and targeted support for vulnerable populations. Furthermore, strengthening healthcare infrastructure and dismantling systemic barriers are crucial measures to reduce mortality rates and improve public health outcomes.

## Supplementary Information

Supplementary Material 1.

Supplementary Material 2.

## Data Availability

Data is available in the supplemental material.

## Abbreviations

APC: Annual Percentage Change
AAMR: Age-Adjusted Mortality Rate
NH: Non-Hispanic
