# Supplementary material for: Temporal patterns in diabetes and sepsis mortality in older Americans: a population-based analysis
Source: BMC Geriatr. 2026 Jan 26;26:236. doi: 10.1186/s12877-026-06989-8 (PMC12918242; doi:10.1186/s12877-026-06989-8)
Supplement: Supplementary file 1 — Supplementary Material 1. [file 12877_2026_6989_MOESM1_ESM.docx]

**Temporal Patterns in Diabetes and Sepsis Mortality in Older Americans: A Population-Based Analysis**

**Supplementary Figure 1:** Sepsis and diabetes related Joinpoint analysis of race/ethnicity from 1999-2020


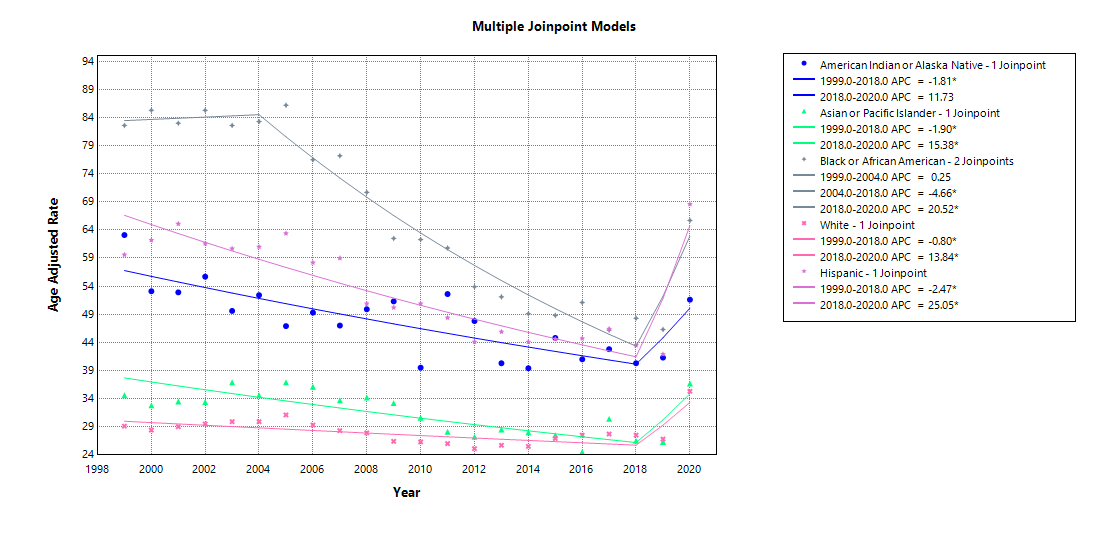


**Supplementary Figure 2:** Sepsis and diabetes related Joinpoint analysis of census region from 1999-2020


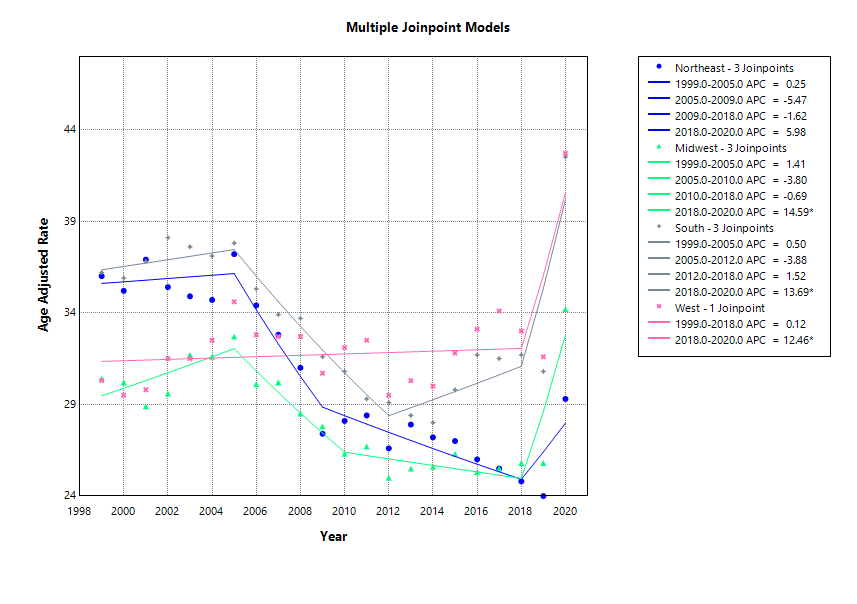


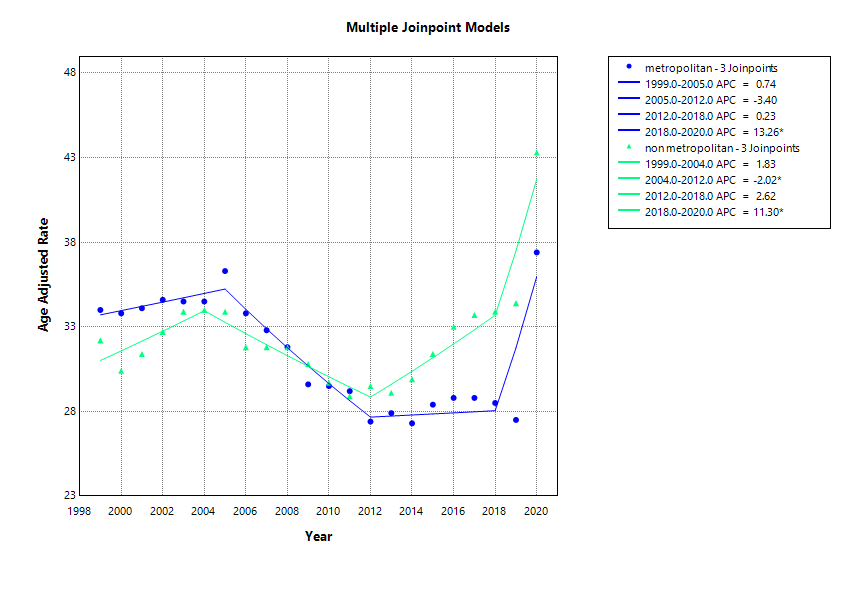
**Supplementary Figure 3:** Sepsis and diabetes related Joinpoint analysis of urbanization from 1999-2020


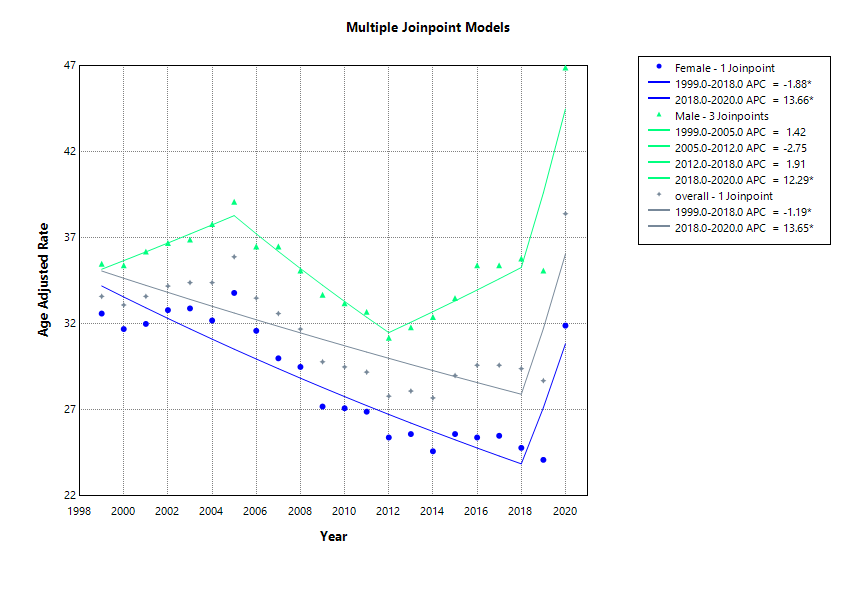
**Supplementary Figure 4:** Sepsis and diabetes related Joinpoint analysis of gender and overall population from 1999-2020

**Supplementary Table 1:** Sepsis and diabetes related Deaths, stratified by place of death from 1999 to 2020

| **Year** | **Medical Facility** | **Nursing Home/Long-term Care Facility** | **Hospices** | **Descendant Home** |
| --- | --- | --- | --- | --- |
| 1999 | 9063 | 2078 | missing | 382 |
| 2000 | 9019 | 2061 | missing | 397 |
| 2001 | 9321 | 2012 | missing | 396 |
| 2002 | 9771 | 1860 | missing | 407 |
| 2003 | 9734 | 2002 | 10 | 443 |
| 2004 | 9926 | 1864 | 26 | 473 |
| 2005 | 10387 | 2014 | 69 | 539 |
| 2006 | 9964 | 1784 | 127 | 466 |
| 2007 | 9940 | 1626 | 180 | 505 |
| 2008 | 9724 | 1520 | 246 | 500 |
| 2009 | 9018 | 1534 | 291 | 511 |
| 2010 | 9379 | 1461 | 323 | 530 |
| 2011 | 9339 | 1482 | 413 | 623 |
| 2012 | 9057 | 1476 | 514 | 578 |
| 2013 | 9244 | 1537 | 556 | 704 |
| 2014 | 9481 | 1511 | 674 | 730 |
| 2015 | 10134 | 1571 | 853 | 781 |
| 2016 | 10469 | 1608 | 957 | 888 |
| 2017 | 10924 | 1591 | 978 | 898 |
| 2018 | 11089 | 1621 | 1020 | 1012 |
| 2019 | 11053 | 1556 | 1114 | 1040 |
| 2020 | 15649 | 1779 | 1233 | 1660 |

**Supplementary Table 2:** Overall and Sex‐Stratified age-adjusted rate from 1999-2020

| Year | Men | Women | Overall |
| --- | --- | --- | --- |
| 1999 | 35.5 (34.5–36.6) | 32.6 (31.8–33.4) | 33.6 (33.0–34.2) |
| 2000 | 35.4 (34.4–36.4) | 31.7 (30.9–32.5) | 33.1 (32.5–33.7) |
| 2001 | 36.2 (35.2–37.2) | 32.0 (31.2–32.7) | 33.6 (33.0–34.2) |
| 2002 | 36.7 (35.6–37.7) | 32.8 (32.1–33.6) | 34.2 (33.6–34.8) |
| 2003 | 36.9 (35.9–37.9) | 32.9 (32.2–33.7) | 34.4 (33.8–35.0) |
| 2004 | 37.8 (36.8–38.8) | 32.2 (31.5–33.0) | 34.4 (33.8–35.0) |
| 2005 | 39.1 (38.1–40.1) | 33.8 (33.0–34.5) | 35.9 (35.3–36.5) |
| 2006 | 36.5 (35.5–37.5) | 31.6 (30.9–32.4) | 33.5 (32.9–34.0) |
| 2007 | 36.5 (35.5–37.5) | 30.0 (29.2–30.7) | 32.6 (32.0–33.1) |
| 2008 | 35.1 (34.2–36.0) | 29.5 (28.8–30.2) | 31.7 (31.2–32.3) |
| 2009 | 33.7 (32.8–34.6) | 27.2 (26.5–27.9) | 29.8 (29.3–30.3) |
| 2010 | 33.2 (32.3–34.0) | 27.1 (26.5–27.8) | 29.5 (29.0–30.1) |
| 2011 | 32.7 (31.8–33.5) | 26.9 (26.2–27.6) | 29.2 (28.7–29.8) |
| 2012 | 31.2 (30.4–32.1) | 25.4 (24.8–26.1) | 27.8 (27.3–28.3) |
| 2013 | 31.8 (30.9–32.6) | 25.6 (25.0–26.2) | 28.1 (27.6–28.6) |
| 2014 | 32.4 (31.6–33.2) | 24.6 (24.0–25.2) | 27.7 (27.2–28.2) |
| 2015 | 33.5 (32.7–34.3) | 25.6 (25.0–26.2) | 29.0 (28.5–29.5) |
| 2016 | 35.4 (34.5–36.2) | 25.4 (24.8–26.0) | 29.6 (29.1–30.1) |
| 2017 | 35.4 (34.5–36.2) | 25.5 (24.9–26.1) | 29.6 (29.1–30.1) |
| 2018 | 35.8 (35.0–36.6) | 24.8 (24.2–25.4) | 29.4 (29.0–29.9) |
| 2019 | 35.1 (34.3–35.8) | 24.1 (23.5–24.6) | 28.7 (28.2–29.1) |
| 2020 | 46.9 (46.1–47.8) | 31.9 (31.3–32.6) | 38.4 (37.9–38.9) |

**Supplementary Table 3:** Race/ethnicity stratified age-adjusted rate from 1999-2020

| **Age-Adjusted Rate (95% CI)** | | | | | |
| --- | --- | --- | --- | --- | --- |
| **Year** | **White** | **Black or African American** | **American Indian or Alaska Native** | **Hispanic or Latino** | **Asian or Pacific Islander** |
| **1999** | 29.1 (28.5–29.7) | 82.6 (79.2–86.0) | 63.1 (50.2–78.3) | 59.6 (55.6–63.5) | 34.6 (30.1–39.0) |
| **2000** | 28.4 (27.8–29.0) | 85.3 (81.9–88.8) | 53.1 (42.0–66.2) | 62.2 (58.2–66.2) | 32.8 (28.6–36.9) |
| **2001** | 29.0 (28.4–29.6) | 83.0 (79.6–86.3) | 52.9 (42.0–65.9) | 65.1 (61.2–69.0) | 33.5 (29.5–37.6) |
| **2002** | 29.5 (28.9–30.1) | 85.3 (81.9–88.7) | 55.7 (44.6–68.8) | 61.6 (57.9–65.4) | 33.4 (29.5–37.3) |
| **2003** | 29.9 (29.3–30.5) | 82.6 (79.3–85.9) | 49.6 (39.5–61.6) | 60.7 (57.1–64.2) | 36.9 (32.9–40.8) |
| **2004** | 29.9 (29.3–30.5) | 83.3 (80.0–86.7) | 52.4 (42.0–64.6) | 61.0 (57.5–64.5) | 34.6 (30.8–38.3) |
| **2005** | 31.1 (30.5–31.7) | 86.2 (82.8–89.5) | 46.9 (37.4–58.1) | 63.4 (59.9–66.9) | 36.9 (33.1–40.6) |
| **2006** | 29.3 (28.7–29.9) | 76.5 (73.4–79.6) | 49.3 (39.8–60.5) | 58.2 (54.9–61.5) | 36.1 (32.5–39.6) |
| **2007** | 28.3 (27.8–28.9) | 77.2 (74.1–80.3) | 47.0 (38.0–57.6) | 59.0 (55.8–62.2) | 33.7 (30.3–37.0) |
| **2008** | 27.9 (27.4–28.5) | 70.7 (67.8–73.6) | 49.9 (40.2–59.7) | 50.9 (48.0–53.8) | 34.2 (30.9–37.5) |
| **2009** | 26.4 (25.8–26.9) | 62.5 (59.8–65.2) | 51.3 (41.8–60.7) | 50.2 (47.4–53.0) | 33.2 (30.0–36.3) |
| **2010** | 26.3 (25.8–26.8) | 62.3 (59.6–64.9) | 39.5 (31.6–48.7) | 50.9 (48.1–53.6) | 30.6 (27.6–33.6) |
| **2011** | 26.0 (25.5–26.6) | 60.8 (58.2–63.4) | 52.6 (43.4–61.9) | 48.4 (45.8–51.0) | 28.1 (25.4–30.9) |
| **2012** | 25.1 (24.6–25.6) | 53.9 (51.5–56.3) | 47.8 (39.3–56.2) | 44.1 (41.7–46.5) | 27.2 (24.6–29.8) |
| **2013** | 25.7 (25.1–26.2) | 52.1 (49.8–54.4) | 40.3 (32.7–47.8) | 45.9 (43.5–48.3) | 28.5 (26.0–31.1) |
| **2014** | 25.5 (25.0–26.0) | 49.1 (46.9–51.3) | 39.4 (32.1–46.8) | 44.1 (41.8–46.4) | 28.0 (25.5–30.4) |
| **2015** | 26.9 (26.4–27.4) | 48.8 (46.7–51.0) | 44.8 (37.2–52.4) | 44.6 (42.4–46.8) | 27.5 (25.2–29.9) |
| **2016** | 27.5 (27.0–28.0) | 51.1 (48.9–53.2) | 41.0 (34.0–48.0) | 44.7 (42.5–46.9) | 24.5 (22.3–26.6) |
| **2017** | 27.7 (27.2–28.2) | 46.2 (44.2–48.2) | 42.8 (35.9–49.6) | 46.4 (44.2–48.5) | 30.4 (28.1–32.7) |
| **2018** | 27.5 (27.0–28.0) | 48.3 (46.3–50.4) | 40.3 (33.9–46.7) | 43.5 (41.4–45.5) | 26.5 (24.4–28.6) |
| **2019** | 26.8 (26.3–27.3) | 46.3 (44.4–48.3) | 41.3 (34.9–47.7) | 41.9 (39.9–43.8) | 26.3 (24.3–28.3) |
| **2020** | 35.3 (34.8–35.9) | 65.7 (63.4–67.9) | 51.6 (44.8–58.5 | 68.6 (66.2–71.1) | 36.7 (34.4–39.1) |

**Supplementary Table 4:** Census region stratified age-adjusted rate from 1999- 2020

| **Year** | **Northeast** | **Midwest** | **South** | **West** |
| --- | --- | --- | --- | --- |
| **1999** | 36.0 (34.7–37.4) | 30.4 (29.2–31.6) | 36.2 (35.1–37.3) | 30.3 (29.0–31.6) |
| **2000** | 35.2 (33.8–36.5) | 30.2 (29.1–31.4) | 35.9 (34.8–36.9) | 29.5 (28.2–30.8) |
| **2001** | 36.9 (35.5–38.2) | 28.9 (27.8–30.1) | 36.8 (35.7–37.8) | 29.8 (28.5–31.1) |
| **2002** | 35.4 (34.0–36.7) | 29.6 (28.5–30.8) | 38.1 (37.0–39.2) | 31.5 (30.2–32.8) |
| **2003** | 34.9 (33.6–36.2) | 31.7 (30.5–32.9) | 37.6 (36.5–38.6) | 31.5 (30.2–32.8) |
| **2004** | 34.7 (33.4–36.0) | 31.6 (30.4–32.7) | 37.1 (36.0–38.1) | 32.5 (31.2–33.8) |
| **2005** | 37.2 (35.9–38.6) | 32.7 (31.5–34.0) | 37.8 (36.8–38.9) | 34.6 (33.3–35.9) |
| **2006** | 34.4 (33.1–35.7) | 30.1 (29.0–31.3) | 35.3 (34.3–36.3) | 32.8 (31.5–34.1) |
| **2007** | 32.8 (31.5–34.0) | 30.2 (29.1–31.4) | 33.9 (32.9–34.9) | 32.7 (31.5–34.0) |
| **2008** | 31.0 (29.7–32.2) | 28.5 (27.4–29.6) | 33.7 (32.8–34.7) | 32.7 (31.5–34.0) |
| **2009** | 27.4 (26.2–28.6) | 27.8 (26.7–28.9) | 31.6 (30.7–32.6) | 30.7 (29.5–31.9) |
| **2010** | 28.1 (26.9–29.2) | 26.3 (25.3–27.4) | 30.8 (29.9–31.7) | 32.1 (30.9–33.3) |
| **2011** | 28.4 (27.2–29.5) | 26.7 (25.6–27.7) | 29.3 (28.4–30.2) | 32.5 (31.3–33.6) |
| **2012** | 26.6 (25.5–27.7) | 25.0 (24.0–26.0) | 29.1 (28.3–30.0) | 29.5 (28.4–30.7) |
| **2013** | 27.9 (26.8–29.1) | 25.5 (24.5–26.5) | 28.4 (27.6–29.2) | 30.3 (29.2–31.4) |
| **2014** | 27.2 (26.1–28.3) | 25.6 (24.6–26.6) | 28.0 (27.2–28.8) | 30.0 (28.9–31.1) |
| **2015** | 27.0 (25.9–28.1) | 26.3 (25.3–27.3) | 29.8 (28.9–30.6) | 31.8 (30.7–33.0) |
| **2016** | 26.0 (25.0–27.1) | 25.3 (24.4–26.3) | 31.7 (30.9–32.6) | 33.1 (32.0–34.2) |
| **2017** | 25.5 (24.5–26.5) | 25.5 (24.5–26.5) | 31.5 (30.7–32.3) | 34.1 (33.0–35.2) |
| **2018** | 24.8 (23.8–25.8) | 25.8 (24.9–26.8) | 31.7 (30.9–32.5) | 33.0 (31.9–34.1) |
| **2019** | 24.0 (23.1–25.0) | 25.8 (24.9–26.8) | 30.8 (30.0–31.6) | 31.6 (30.6–32.7) |
| **2020** | 29.3 (28.2–30.3) | 34.2 (33.1–35.3) | 42.5 (41.6–43.4) | 42.7 (41.5–43.9) |

**Supplementary Table 5:** AAMR trends stratified by urbanization

| **Age-adjusted rate** | | |
| --- | --- | --- |
| **Year** | **Metropolitan** | **Non-metropolitan** |
| **1999** | 34.0 (33.3–34.7) | 32.2 (30.8–33.5) |
| **2000** | 33.8 (33.1–34.5) | 30.4 (29.1–31.7) |
| **2001** | 34.1 (33.4–34.7) | 31.4 (30.1–32.8) |
| **2002** | 34.6 (33.9–35.3) | 32.7 (31.3–34.0) |
| **2003** | 34.5 (33.8–35.2) | 33.9 (32.5–35.3) |
| **2004** | 34.5 (33.8–35.1) | 34.0 (32.7–35.4) |
| **2005** | 36.3 (35.6–37.0) | 33.9 (32.5–35.2) |
| **2006** | 33.8 (33.1–34.4) | 31.8 (30.5–33.1) |
| **2007** | 32.8 (32.1–33.4) | 31.8 (30.5–33.1) |
| **2008** | 31.8 (31.1–32.4) | 31.8 (30.5–33.2) |
| **2009** | 29.6 (29.0–30.2) | 30.8 (29.5–32.0) |
| **2010** | 29.5 (28.9–30.0) | 29.7 (28.4–30.9) |
| **2011** | 29.2 (28.7–29.8) | 28.9 (27.7–30.1) |
| **2012** | 27.4 (26.8–27.9) | 29.5 (28.3–30.7) |
| **2013** | 27.9 (27.3–28.5) | 29.1 (27.9–30.3) |
| **2014** | 27.3 (26.8–27.8) | 29.9 (28.7–31.1) |
| **2015** | 28.4 (27.9–28.9) | 31.4 (30.2–32.6) |
| **2016** | 28.8 (28.3–29.4) | 33.0 (31.7–34.2) |
| **2017** | 28.8 (28.2–29.3) | 33.7 (32.4–34.9) |
| **2018** | 28.5 (28.0–29.0) | 33.9 (32.7–35.2) |
| **2019** | 27.5 (27.0–28.0) | 34.4 (33.2–35.6) |
| **2020** | 37.4 (36.8–38.0) | 43.3 (41.9–44.6) |

**Supplementary Table 6:** APC trends stratified by sex, race, urbanization, and census

| Year | Overall | Women | Men | White | Black or African American | Asian or Pacific Islander | American Indian or Alaska Native | Hispanic or Latino |
| --- | --- | --- | --- | --- | --- | --- | --- | --- |
| 1999 | 11631 | 6875 | 4756 | 9007 | 2293 | 245 | 86 | 915 |
| 2000 | 11560 | 6762 | 4798 | 8840 | 2388 | 251 | 81 | 983 |
| 2001 | 11850 | 6850 | 5000 | 9133 | 2361 | 273 | 83 | 1098 |
| 2002 | 12189 | 7072 | 5117 | 9350 | 2449 | 300 | 90 | 1094 |
| 2003 | 12401 | 7141 | 5260 | 9567 | 2407 | 341 | 86 | 1138 |
| 2004 | 12520 | 7050 | 5470 | 9636 | 2453 | 338 | 93 | 1205 |
| 2005 | 13245 | 7470 | 5775 | 10171 | 2599 | 386 | 89 | 1315 |
| 2006 | 12528 | 7050 | 5478 | 9678 | 2351 | 402 | 97 | 1263 |
| 2007 | 12426 | 6803 | 5623 | 9517 | 2418 | 392 | 99 | 1327 |
| 2008 | 12356 | 6812 | 5544 | 9548 | 2283 | 419 | 106 | 1216 |
| 2009 | 11791 | 6358 | 5433 | 9168 | 2070 | 434 | 119 | 1252 |
| 2010 | 11887 | 6439 | 5448 | 9274 | 2104 | 417 | 92 | 1316 |
| 2011 | 12071 | 6524 | 5547 | 9397 | 2127 | 415 | 132 | 1346 |
| 2012 | 11869 | 6358 | 5511 | 9335 | 1972 | 432 | 130 | 1304 |
| 2013 | 12305 | 6513 | 5792 | 9732 | 1974 | 483 | 116 | 1433 |
| 2014 | 12547 | 6421 | 6126 | 9967 | 1948 | 514 | 118 | 1466 |
| 2015 | 13451 | 6895 | 6556 | 10753 | 2013 | 543 | 142 | 1586 |
| 2016 | 14093 | 6985 | 7108 | 11235 | 2207 | 509 | 142 | 1664 |
| 2017 | 14580 | 7192 | 7388 | 11659 | 2080 | 681 | 160 | 1832 |
| 2018 | 14898 | 7202 | 7696 | 11841 | 2260 | 637 | 160 | 1804 |
| 2019 | 14945 | 7139 | 7806 | 11860 | 2257 | 659 | 169 | 1823 |
| 2020 | 20595 | 9717 | 10878 | 16035 | 3346 | 980 | 234 | 3167 |

**Supplementary Table 7:** Sepsis and diabetes related Deaths, stratified by Sex and Race, in Adults in the United

| Variable | Trend | Year | APC (95% CI) |
| --- | --- | --- | --- |
|  | Segment |  |  |
| OVERALL POPULATION | 1 | 1999-2018 | -1.19* (-2.18 to -0.67) |
|  | 2 | 2018-2020 | 13.65* (1.35 to 19.77) |
| SEX |  |  |  |
| Male | 1 | 1999-2005 | 1.42 (-4.46 to 11.52) |
|  | 2 | 2005-2012 | -2.75 (-7.96 to 5.82) |
|  | 3 | 2012-2018 | 1.91 (-3.71 to 5.27) |
|  | 4 | 2018-2020 | 12.29* (3.49 to 17.69) |
| Female | 1 | 1999-2018 | -1.88* (-2.47 to -1.41) |
|  | 2 | 2018-2020 | 13.66* (3.42 to 18.71) |
|  |  |  |  |
| METROPOLITAN | 1 | 1999-2005 | 0.74 (-3.79 to 6.95) |
|  | 2 | 2005-2012 | -3.40 (-7.74 to 3.95) |
|  | 3 | 2012-2018 | 0.23 (-4.16 to 3.66) |
|  | 4 | 2018-2020 | 13.26* (4.06 to 19.03) |
| NON-METROPOLITAN | 1 | 1999-2004 | 1.83 (-0.10 to 8.75) |
|  | 2 | 2004-2012 | -2.02* (-6.18 to -0.82) |
|  | 3 | 2012-2018 | 2.62 (-1.38 to 5.05) |
|  | 4 | 2018-2020 | 11.30* (4.88 to 15.27) |
| Northeast | 1 | 1999-2005 | 0.25 (-2.86 to 3.70) |
|  | 2 | 2005-2009 | -5.47 (-8.16 to 2.10) |
|  | 3 | 2009-2018 | -1.62 (-5.09 to 0.05) |
|  | 4 | 2018-2020 | 5.98 (-0.79 to 9.57) |
| Midwest | 1 | 1999-2005 | 1.41 (-0.33 to 5.06) |
|  | 2 | 2005-2010 | -3.80 (-7.13 to 0.21) |
|  | 3 | 2010-2018 | -0.69 (-2.49 to 1.86) |
|  | 4 | 2018-2020 | 14.59* (7.12 to 19.01) |
| South | 1 | 1999-2005 | 0.50 (-4.67 to 9.49) |
|  | 2 | 2005-2012 | -3.88 (-9.39 to 4.40) |
|  | 3 | 2012-2018 | 1.52 (-4.31 to 5.21) |
|  | 4 | 2018-2020 | 13.69* (3.87 to 19.64) |
| West | 1 | 1999-2018 | 0.12 (-0.76 to 0.67) |
|  | 2 | 2018-2020 | 12.46* (2.50 to 17.61) |
| American Indian or Alaska Native | 1 | 1999-2018 | -1.81* (-4.24 to -0.87) |
|  | 2 | 2018-2020 | 11.73 (-0.94 to 18.60) |
| Asian or Pacific Islander | 1 | 1999-2018 | -1.90* (-3.69 to -0.96) |
|  | 2 | 2018-2020 | 15.38* (0.47 to 23.10) |
| Black or African American | 1 | 1999-2004 | 0.25 (-2.65 to 7.78) |
|  | 2 | 2004-2018 | -4.66* (-6.37 to -4.06) |
|  | 3 | 2018-2020 | 20.52* (9.56 to 27.79) |
| White | 1 | 1999-2018 | -0.80* (-1.99 to -0.26) |
|  | 2 | 2018-2020 | 13.84* (1.41 to 20.24) |
| Hispanic | 1 | 1999-2018 | -2.47* (-3.38 to -1.68) |
|  | 2 | 2018-2020 | 25.05* (10.46 to 32.98) |
